# Psychiatric co-morbidity and substance abuse after gastric bypass surgery

**DOI:** 10.1093/bjs/znad179

**Published:** 2023-06-14

**Authors:** Carl Johan Svensson, Kok Wai Giang, John Wallert, Christian Rück, Christina E Lundberg

**Affiliations:** Department of Anaesthesia, Operation & Intensive Care, Sahlgrenska University Hospital, Region Västra Götaland, Gothenburg, Sweden; Department of Anaesthesiology and Intensive Care Medicine, Institute of Clinical Sciences at the Sahlgrenska Academy, University of Gothenburg, Gothenburg, Sweden; Department of Molecular and Clinical Medicine, Sahlgrenska Academy, University of Gothenburg, Gothenburg, Sweden; Department of Medicine, Geriatrics and Emergency Medicine/Östra, Sahlgrenska University Hospital/Östra, Region Västra Götaland, Gothenburg, Sweden; Centre for Psychiatry Research, Department of Clinical Neuroscience, Karolinska Institutet, & Stockholm Healthcare Services, Region Stockholm, Huddinge, Sweden; Centre for Psychiatry Research, Department of Clinical Neuroscience, Karolinska Institutet, & Stockholm Healthcare Services, Region Stockholm, Huddinge, Sweden; Department of Molecular and Clinical Medicine, Sahlgrenska Academy, University of Gothenburg, Gothenburg, Sweden; Department of Food and Nutrition, and Sport Science, University of Gothenburg, Gothenburg, Sweden

## Introduction

Bariatric surgery^1,2^ may reduce the incidence of depressive disorders in patients with obesity^3–5^. However, there are several potential adverse postoperative side effects, both physical (for example, malnutrition, gallstone development and bowel obstruction)^1^ and behavioural (for example, alcohol-related disorders and opioid use)^6–8^. Moreover, the rate of self-harm and suicide attempts are higher in this patient group compared with the general population^6,9^ and, possibly, non-operated patients with obesity^10,11^. Also, a long-term cohort study found more major depression in obese patientes of both sexes after gastric bypass compared to restrictive surgery^12^.

Several studies have investigated the association between gastric bypass and psychiatric disorders^3,5,6^ but none has compared those risks with non-operated patients with obesity and matched non-obese population controls. In the current study, we used the Swedish Patient Registry to investigate all 71,488 patients (20-65 years) with a principal diagnosis of obesity between 2001–2013, and compared the long-term incidence of psychiatric co-morbidities in gastric bypass patients with non-operated patients with obesity (and matched population controls without obesity).

## Methods

### Study design

In this nationwide registry-based cohort study, we included all individuals aged 20–65 years with a first recorded principal diagnosis of obesity (ICD-10 codes E65 or E66) in the Patient Registry between 1 January 2001 and 31 December 2013. For each patient with obesity, two control participants, matched by year of birth, sex and area of residence, and without an obesity diagnosis or bariatric surgery code, were randomly selected from Sweden’s Registry of the Total Population. Outcomes compared between gastric bypass patients and non-operated patients with obesity and matched population controls included depressive disorders, anti-depression medications, neurotic, stress-related and somatoform disorders, alcohol-related disorders, other substance use disorders and suicide.

Detailed information on methodology, including data acquisition, inclusions and exclusions, and bias adjustments have been published previously^13^. See *Supplementary material* for a description of the methods specific to this study, including codes from the NOMESCO classification of surgical procedures (*Table S1*), codes from the international definition of diseases (*Table S2*), definition of co-morbidity and outcomes, bias adjustments and statistical analysis, and study limitations.

## Results

### Participants

Among all patients with an obesity diagnosis, 30 214 individuals underwent gastric surgery surgery, of which 28 204 remained after exclusions (*Fig. S1*). There were 41 274 non-operated individuals with an obesity diagnosis, of which 40 827 remained after exclusions (*Fig. S1*). Gastric bypass patients and non-operated individuals with an obesity diagnosis were matched with 55 903 and 80 800 population controls, respectively. *Table 1* presents baseline study population characteristics. *Table 2* presents events, median follow-up times and incidence rates for all outcomes and study groups.

**Table 1:** Characteristics for gastric bypass patients, non-operated patients with obesity and the two matched population control groups without obesity

	Gastric bypass patients	Non-obese population controls	Non-operated patients with obesity	Non-obese population controls
**Number**	**28 204**	**55 903**	**40 827**	**80 800**
***Baseline***				
Age (years), mean(s.d.)	40.8(10.4)	40.8(10.4)	43.2(11.8)	43.1(11.7)
**Age group, *n* (%)**				
20–44	17 859 (63.5)	35 480 (63.6)	22 253 (55.2)	44 233 (55.4)
45–54	7207 (25.6)	14 252 (25.6)	10 039 (24.9)	19 872 (24.9)
55–64	3070 (10.9)	6038 (10.8)	8030 (19.9)	15 720 (19.7)
**Sex, *n* (%)**				
Women	21 295 (75.5)	42 203 (75.5)	27 983 (68.5)	55 460 (68.6)
Education level*, *n* (%)				
9 years or less	4859 (17.2)	6574 (11.8)	8432 (20.7)	11 654 (14.4)
10–12 years	17 327 (61.4)	25 354 (45.4)	22 002 (53.9)	36 876 (45.6)
>12 years	5896 (20.9)	23 160 (41.4)	9987 (24.5)	31 157 (38.6)
**Marital status^†^, *n* (%)**				
Single	11 651 (41.3)	23 862 (42.7)	15 642 (38.3)	31 242 (38.7)
Married/registered partner	12 011 (42.6)	24 348 (43.6)	17 563 (43.0)	36 791 (45.5)
Divorced/widowed	4541 (16.1)	7683 (13.7)	7621 (18.7)	12 746 (15.8)
**Psychiatric history, *n* (%)**				
Depressive disorders (diagnosis)	3756 (13.3)	3159 (5.7)	5081 (12.4)	3729 (4.6)
Antidepressant medication^‡^	10 903 (38.7)	11 128 (19.9)	12 920 (31.6)	12 206 (15.1)
Neurotic, stress-related and somatoform disorders	4150 (14.7)	4379 (7.8)	6276 (15.4)	5152 (6.4)
Alcohol-related disorders	1012 (3.6)	1493 (2.7)	1719 (4.2)	1978 (2.4)
Other substance use disorders	940 (3.3)	886 (1.6)	1702 (4.2)	1154 (1.4)
**Co-morbidity, *n* (%)**				
Hypertension	11 157 (39.6)	7880 (14.1)	16 490 (40.4)	11 612 (14.4)
Diabetes mellitus	5105 (18.1)	1448 (2.6)	8392 (20.6)	2434 (3.0)
Cardiovascular disease	1011 (3.6)	768 (1.4)	2870 (7.0)	1777 (2.2)
Malignancy	759 (2.7)	1734 (3.1)	1900 (4.7)	3063 (3.8)

Missing education, *n* = 2456 (1.2 per cent). †Missing marital status, *n* = 33 (0.0 per cent). ‡Dispensed prescription from the pharmacotherapeutic group N06A (according to the Anatomical Therapeutic Chemical classification system, ATC), 2005 and onwards.

**Table 2:** Numbers, events, follow-up times and incidence rates for all psychiatric outcomes

Outcomes	*N*	Event (%)	Median follow-up time (i.q.r.)	Incidence rates (95% c.i.)
**Depressive disorders (diagnosis) and neurotic, stress-related and somatoform disorders**				
Gastric bypass patients	16 322	1149 (7.0)	4.0 (2.5, 5.8)	15.0 (14.1–15.9)
Non-obese population controls	43 107	1181 (2.7)	4.1 (2.6, 6.0)	5.6 (5.3–6.0)
Non-operated obese patients	25 698	2008 (7.8)	5.1 (2.5, 8.9)	15.0 (14.3–15.7)
Non-obese population controls	66 007	2420 (3.7)	5.0 (2.5, 8.8)	6.9 (6.6–7.2)
**Depressive disorders (diagnosis)**				
Gastric bypass patients	16 932	656 (3.9)	4.1 (2.6, 5.9)	8.1 (7.5–8.8)
Non-obese population controls	44 257	541 (1.2)	4.1 (2.7, 6.1)	2.5 (2.3–2.7)
Non-operated obese patients	26 966	1330 (4.9)	5.3 (2.7, 9.3)	9.1 (8.6–9.6)
Non-obese population controls	67 640	1333 (2.0)	5.1 (2.6, 8.9)	3.7 (3.5–3.9)
**Neurotic, stress-related and somatoform disorders**				
Gastric bypass patients	24 054	2027 (8.4)	3.9 (2.4, 5.7)	18.6 (17.8–19.4)
Non-obese population controls	51 524	1687 (3.3)	4.0 (2.6, 5.9)	6.9 (6.6–7.2)
Non-operated obese patients	34 551	2454 (7.1)	4.7 (2.3, 8.2)	14.5 (13.9–15.1)
Non-obese population controls	75 648	2591 (3.4)	4.8 (2.4, 8.4)	6.6 (6.4–6.9)
**Antidepressant medication**				
Gastric bypass patients	16 156	3180 (19.7)	3.5 (2.1, 5.2)	49.9 (48.2–51.8)
Non-obese population controls	42 617	3727 (8.7)	3.9 (2.3, 5.6)	20.2 (19.5–20.9)
Non-operated obese patients	19 853	3717 (18.7)	3.8 (1.9, 6.3)	45.8 (44.3–47.3)
Non-obese population controls	52 813	5248 (9.9)	4.0 (2.1, 6.6)	22.8 (22.2–23.5)
**Alcohol-related disorders**				
Gastric bypass patients	27 192	1243 (4.6)	3.9 (2.5, 5.8)	10.8 (10.2–11.5)
Non-obese population controls	54 410	404 (0.7)	4.1 (2.6, 5.9)	1.7 (1.6–1.9)
Non-operated obese patients	39 108	630 (1.7)	4.8 (2.4, 8.3)	3.1 (2.8–3.3)
Non-obese population controls	78 822	681 (0.9)	4.9 (2.4, 8.6)	1.6 (1.5–1.7)
**Other substance use disorders**				
Gastric bypass patients	27 264	793 (2.9)	4.0 (2.5; 5.8)	6.4 (5.9–6.8)
Non-obese population controls	55 017	380 (0.7)	4.1 (2.6; 5.9)	1.6 (1.4–1.8)
Non-operated obese patients	39 017	816 (2.1)	4.8 (2.4; 8.3)	4 (3.7–4.3)
Non-obese population controls	79 646	646 (0.8)	4.9 (2.4; 8.6)	1.5 (1.4–1.6)
**Suicide**				
Gastric bypass patients	28 204	98 (0.3)	4.1 (2.6; 5.9)	0.8 (0.6–1.0)
Non-obese population controls	55 903	39 (0.1)	4.1 (2.6; 5.9)	0.2 (0.1–0.2)
Non-operated obese patients	40 827	94 (0.2)	4.8 (2.4; 8.4)	0.4 (0.4–0.5)
Non-obese population controls	80 800	64 (0.1)	4.9 (2.4; 8.6)	0.1 (0.1–0.2)

c.i.: confidence intervals; IQR: Inter.

### Gastric bypass patients *versus* non-operated patients with obesity *versus* population controls

Gastric bypass patients were younger and included a higher proportion of women compared to non-operated patients with obesity (*Table 1*). Individuals diagnosed with obesity had a higher baseline prevalence of psychiatric co-morbidities compared with matched population controls (*Table 1*).

**Fig. 1:**
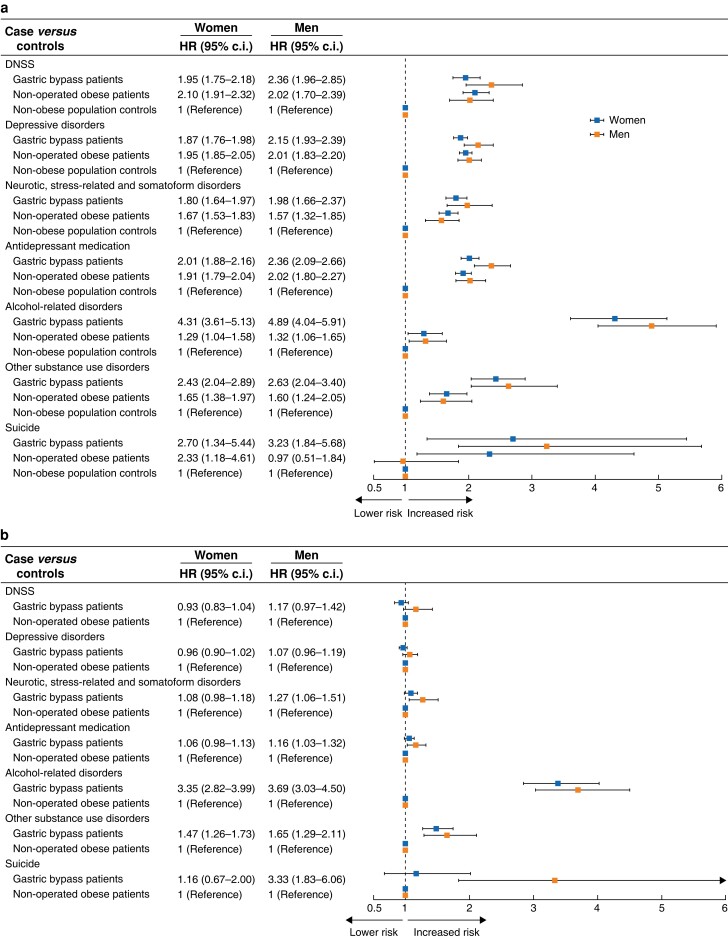
Hazard ratios Hazard ratios for all outcomes by men and women, comparing **a** gastric bypass patients and non-operated patients with obesity with non-obese population controls and **b** gastric bypass patients with non-operated patients with obesity

Patients with obesity (operated and non-operated) of both sexes had almost twice the risk of being diagnosed with either depressive disorder or neurotic, stress-related and somatoform disorders (DNSS) compared with the general population (*Fig. 1a*, *Table S3*). The risk of DNSS was slightly higher for gastric bypass-operated men compared with non-operated men with obesity (*Fig. 1b*, *Table S4*). Overall, antidepression medication had a similar pattern to DNSS (*Fig. 1a–b*).

The risk of alcohol-related disorders was 4–5 times higher for gastric bypass-operated individuals of both sexes compared with population controls, and 3–4 times higher compared with non-operated patients with obesity (*Fig. 1b*, *Table S4*). The risk was generally higher for men. The risk of substance use disorders showed a similar pattern to alcohol-related disorders but with lower overall risks (*Fig. 1a–b*, *Table S3 and S4*).

The risk of suicide was higher for gastric bypass patients of both sexes compared with population controls and compared with non-operated patients with obesity (*Fig. 1a–b*, Table S3), but the overall incidence rates were very low (*Table 2*). In non-operated patients, only women had a higher risk of suicide compared with population controls (*Fig. 1a*, *Table S3*).

## Discussion

This is the first long-term prospective cohort study from Sweden that compares the incidence of all major psychiatric co-morbidities in adult gastric bypass patients with that in non-operated patients with obesity and matched population controls. We show that gastric bypass surgery is associated with substantially increased risk of alcohol-related disorders and moderately increased risk of other types of substance abuse, particularly in men.

In line with previous studies, both depression and DNSS were associated with obesity at baseline in our study groups^14,15^. However, so was male sex, lower education level and being single, and neither these nor other baseline characteristics differed markedly between operated and non-operated patients with obesity. Hence, none of the co-morbidities analysed here was fuelling the very high incidence of alcohol-related disorders observed among gastric bypass patients.

Our study was the first to study DNSS in gastric bypass patients in Sweden. This outcome was more common in gastric bypass patients compared to non-operated patients with obesity, presenting a possible link to the higher incidence of alcohol-related disorders post-surgery. Earlier studies have shown that neurotic disorders are closely associated with alcoholism, such as in younger patients who use alcohol as a way of coping with nervousness and agitation^15,16^. However, studies on siblings and twins suggest that these disorders are often a consequence rather than a cause of alcoholism^17^.

Like others^6,9^, we found a higher risk of suicide among gastric bypass patients compared with non-operated patients with obesity. However, the absolute incidence of suicide was very low (0.1–0.3 per cent) and it is difficult to draw general conclusions based on such sparse data. Other large cohort studies have shown that pre-existing depression and anxiety, male sex and gastric bypass operation led to augmented suicide risk after bariatric surgery^11,18^.

Bypassing the greater part of the stomach, duodenum and parts of the jejunum affect the absorption of ingested food and liquids, including alcohol and other drugs^19,20^. A recent review on the relationship between bariatric surgery and alcohol use discussed a possible mechanism derived from rodent studies, which was that reduced gastric ghrelin secretion may cause malfunction of the growth hormone secretagogin receptor signalling that is involved in appetite control^21^, which then leads to increased central dopamine discharge that promotes alcohol intake and alcohol rewarding behaviours. Bypassing the stomach also leads to a reduction of first-pass metabolism resulting in higher blood alcohol content^19^. This is in part explained by the reduced gastric alcohol dehydrogenase (ADH) activity found after gastric bypass, which can also be seen in healthy females, who have similar gastric emptying times to men but lower ADH activity^22^. These physical aspects can partly explain why gastric bypass patients, and particularly men, have a higher incidence of alcohol-related disorders.

Gastric bypass surgery was strongly associated with increased risk of alcohol-related and other substance use disorders, and potentially suicide, particularly in males. Psychiatric co-morbidity did not explain this pattern; rather, these outcomes were directly associated with gastric bypass surgery, possibly because of changed alcohol and drug absorption and metabolism. The increased risks associated with male sex and gastric bypass surgery deserve improved attention and perhaps also different management before and after surgery.

## Supplementary Material

znad179_Supplementary_Data

## Data Availability

Data are available from the sources stated in the paper on request to the data providers, fulfilling legal and regulatory requirements and with permission from the Swedish Ethical Review Authority.
