# Corrigendum to: Psychiatric co-morbidity and substance abuse after gastric bypass surgery

**DOI:** 10.1093/bjs/znad227

**Published:** 2023-07-24

This is a corrigendum to: Carl Johan Svensson and others, Psychiatric co-morbidity and substance abuse after gastric bypass surgery, *British Journal of Surgery*, 2023; znad179, https://doi.org/10.1093/bjs/znad179

In the originally published version of this manuscript, there was a typographical error in the name of author Kok Wai Giang.

This has been corrected.

