# Supplementary material for: Psychiatric co-morbidity and substance abuse after gastric bypass surgery
Source: Br J Surg. 2023 Jun 14;110(12):1618–22. doi: 10.1093/bjs/znad179 (PMC10638527; doi:10.1093/bjs/znad179)
Supplement: znad179_Supplementary_Data [file znad179_supplementary_data.docx]

**Psychiatric comorbidity and substance abuse after gastric bypass surgery**

Carl Johan Svensson^1,2^, MD PhD, Kok Wai Gang^3,4^ PhD, John Wallert^5^ PhD,

Christian Rück^5^ Prof, Christina E Lundberg^3,6^, PhD MPH

^1^ Department of Anaesthesia, Operation & Intensive Care, Sahlgrenska University Hospital Region Västra Götaland, Gothenburg, Sweden

^2^ Department of Anaesthesiology and Intensive Care Medicine, Institute of Clinical Sciences at the Sahlgrenska Academy, University of Gothenburg, Gothenburg, Sweden

^3^ Department of Molecular and Clinical Medicine, Sahlgrenska Academy, University of

Gothenburg, Gothenburg, Sweden

^4^ Department of Medicine, Geriatrics and Emergency Medicine/Östra, Sahlgrenska University Hospital/Östra, Region Västra Götaland, Gothenburg, Sweden

^5^ Centre for Psychiatry Research, Department of Clinical Neuroscience, Karolinska Institutet, & Stockholm Healthcare Services, Region Stockholm, Huddinge, Sweden

^6^ Department of Food and Nutrition, and Sport Science, University of Gothenburg, Gothenburg, Sweden

**Corresponding author**: Carl Johan Svensson, Email: [johan.carl.svensson@vgregion.se](mailto:johan.carl.svensson@vgregion.se)

ORCID: 0000-0002-8265-9313

Supplementary material - Methods

Inclusion and exclusion criteria

Data on age, sex, discharge diagnoses, surgical procedures, and hospitalization dates were obtained. Information about socioeconomic variables was obtained from the Longitudinal integrated database for health insurance and labour market studies (LISA) (80% coverage).

Non-operated patients with obesity lacks a date of surgery and are therefore at risk during a longer period than gastric bypass patients, giving rise to an immortal time bias. To avoid this, we used landmark analysis, to split up the follow‐up time, and set the study baseline at 2 years after a recorded obesity diagnosis. This timepoint was chosen because the median time from obesity diagnosis to surgery was 1.5 years. By the 2-year landmark, we captured 81% of all individuals who underwent gastric bypass surgery in Sweden during the study period. Time at risk was calculated from this landmark, and all events that occurred before this landmark were considered comorbidities.

Patients with obesity were divided into two groups, one gastric bypass group and one with non-operated patients with obesity (Figure S1). Non-operated patients with obesity who underwent any type of bariatric surgery during the follow-up time were censored at the date of surgery (For specific surgical procedures and codes, see Table S1). Gastric bypass was defined using the NOMESCO codes JDF10 or JDF11. Inclusion and exclusion criteria can be found in Figure S1.

*Outcomes and comorbidities*

Outcomes were obtained from the Patient Registry and the Cause of Death Registry according to the ICD-10. Outcomes were defined by the following ICD-10 codes: 1) depressive disorders by F32–F34 and F38–F39; 2) Neurotic, stress-related and somatoform disorders by F40–F48; 3) Alcohol-related disorders by F10; 4) Other substance use disorders by F11–F19; and 5) Suicide by X60–X84 and Y10–Y34 (including event of undetermined intent). As many patients with mild to moderate depression in Sweden are treated in primary care, not covered by the National Patient Register, we also considered at least one filled prescription of an antidepressant (ATC-code N06A) prescribed by any physician as a proxy for depressive or neurotic disorders. As the Prescribed drug registry started in 2005, this outcome was considered as a separate event, only including individuals with inclusion date from January 1, 2006, and onwards.

Disorders present prior to or at the study baseline included the following: Depressive disorders, Anti-depressive medications, Neurotic, stress-related and somatoform disorders, Alcohol-related disorders, Other substance use disorders, Diabetes, Hypertension, Cardiovascular disease, and Malignancy (definitions in Table S2).

*Statistical analysis*

Participants were followed from the study baseline until (a) any of the above defined events, (b) death, (c) reaching a maximum follow-up of 10 years, or (d) end of the study (December 31, 2016), whichever occurred first. The follow-up time was restricted to a maximum of 10 years to ensure more even follow-up times between groups. Age- and sex-adjusted incidence rates (IR) were calculated per 1000 person-years along with 95% confidence intervals (CIs).

We used multivariable Cox proportional hazards regression to calculate hazard ratios (HR) with 95% CIs to estimate the relative risk of all outcomes. Contrast matrices were used to compare HRs between groups. Education was categorized into three groups (<9, 10–12, and >12 years of formal education) and was included in the models as an interaction term with the study groups. Marital status was dichotomized as “yes” (single/ divorced/ widowed) or “no” (married/ registered partner). Individuals with missing data on either education or marital status (1.2% in total) were excluded from analysis. Outcomes are adjusted for sex, age, education, marital status, and psychiatric comorbidity at baseline (e.g., depressive disorders is adjusted for baseline neurotic, stress-related and somatoform disorders, anti-depressant medication, alcohol-related disorders and other substance use disorders). Because the relative risk was non-proportional during the follow-up period for some of the regression models, we present follow-up times divided into three time-periods: 0–<2, 2–6 and 6–10 years in Table S3 and Table S4. Proportionality was examined using methods based on weighted residuals, and all final models fulfilled the proportionality assumption of the Cox model.

Data management was performed using SAS version 9.4 (SAS Institute, Cary, NC, USA) and analysis in R version 3.6.2. (The R Project for Statistical Computing, Vienna, Austria). All data were pseudonymized before handled by the researcher. The regional ethical review board in Gothenburg approved the study in 2015 (DNR: 579–15).

*Limitations*

This is a retrospective observation study and selection bias among patients eligible for and willing to undergo GB surgery is present and should be considered when interpreting the results. There is also a non-differential misclassification bias as comparisons to non-operated patients with obesity apply only to those requiring specialized care and not to those managed in primary care settings. Moreover, using one filed prescription of an antidepressant as an alternative proxy indication for depression or neurotic disorders is an overestimate, but this is true in all included study groups, and can therefore still be used when comparing groups.

Supplementary material - Definitions

**Definition of hypertension and diabetes trough dispensed antihypertensive and antidiabetic medication**

Hypertension was defined as having a dispensed antihypertensive medication (ATC codes: C02, C03, C07–C09) prior to or at study baseline. Dispensed antihypertensive medication was not used to define hypertension when the individual had a prior diagnosis of:

- Heart failure or cardiomyopathy (ICD-10 codes: I50 or I42) treated with loop-diuretics (ATC code: C03C) or;
- Heart failure (ICD-10 codes: I50) treated with beta-blocker (ATC code: C07AB02, C07AB07, or C07AG02), ACE inhibitor (ATC code: C09A or C09B), or angiotensin II inhibitor (ATC code: C09C) or;
- Atrial fibrillation, flutter, or tachycardia (ICD-10 codes: I47 or I48) treated with a beta-blocker (ATC code: C07), or calcium antagonist (ATC code: C08D).

Diabetes was defined as having a dispensed antidiabetic medication (ATC codes: A10A or A10B).

Supplementary Figures

Figure S1. Flow diagram of the inclusion and exclusion process.


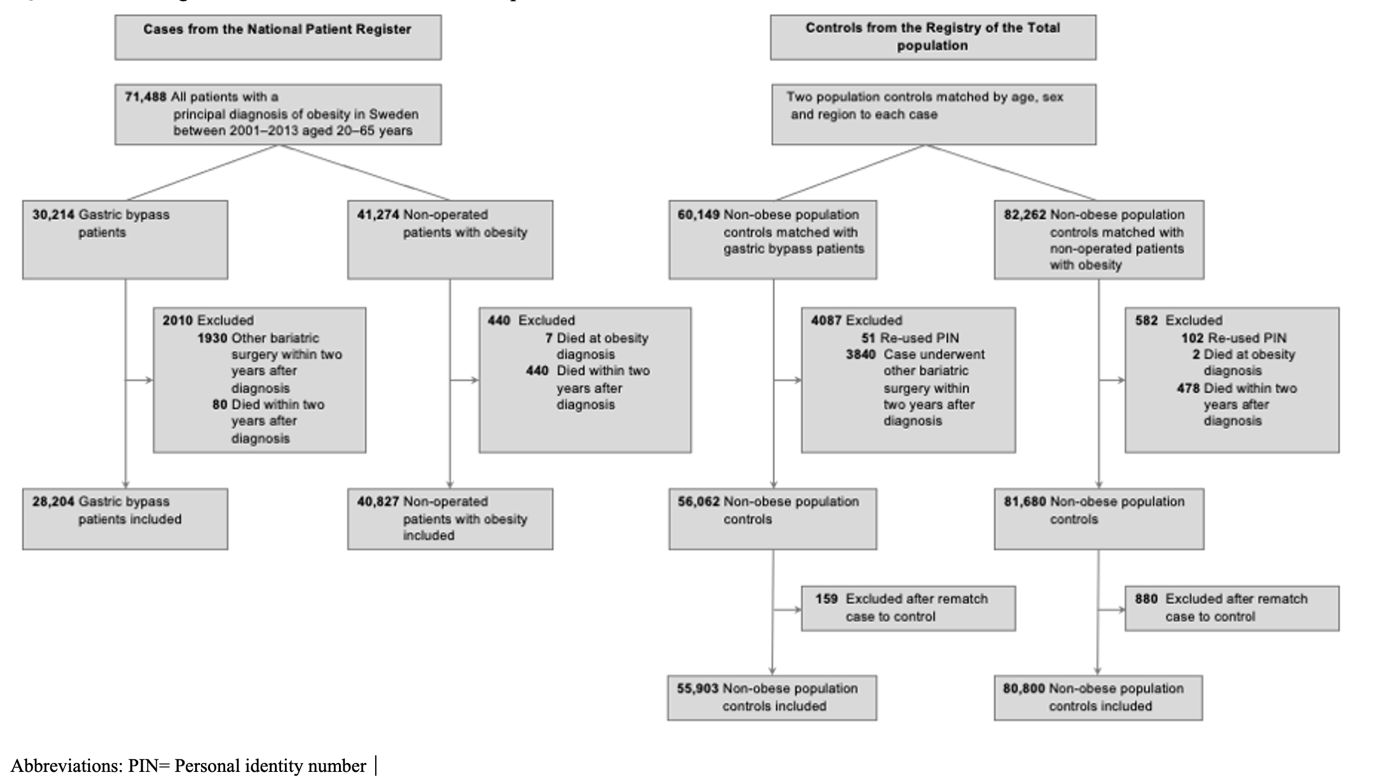


Supplementary Tables

- Table S1. Codes from the Swedish version of the NOMESCO Classification of Surgical Procedures, used for censoring bariatric surgery patients before and during the study period.
- Table S2. Codes from the International Classification of Diseases 9th and 10th Revision, used for censoring and to define comorbidity.
- Table S3. Hazard ratios for all outcomes, divided into three time-periods^a^, comparing gastric bypass patients and non-operated patients with obesity with non-obese population controls.
- Table S4. Hazard ratios for all outcomes, divided into three time-periods^a^, comparing gastric bypass patients with non-operated patients with obesity.

**Table S1. Codes from the Swedish version of the NOMESCO Classification of Surgical Procedures^a^, used for censoring bariatric surgery patients before and during the study period.**

| **Procedure** | **NOMESCO codes** |
| --- | --- |
| Gastric bypass | JDF10, JDF11 |
| Vertical banded gastroplasty | JDF00, JDF01 |
| Gastric banding | JDF20, JDF21 |
| Duodenal swich | JFD03, JFD04 |
| Gastric sleeve | JDF96, JDF97 |

^a^The Swedish version of the NOMESCO Classification of Surgical Procedures (version 1.9) has been in use from 1997 to 2020.

**Table S2. Codes from the International Classification of Diseases 9th and 10th Revision^a^, used for censoring, to define comorbidity and events^b^.**

| **Diagnosis** | **ICD-9** | **ICD-10** |
| --- | --- | --- |
| Depressive disorders^c^ | 300.4, 311 | F32–34, F38–39 |
| Neurotic, stress-related and somatoform disorders | 300.0–3, 300.5–8, 306, 308–9 | F40–F48 |
| Alcohol-related disorders | 291, 303, 305.0 | F10 |
| Other substance use disorders | 292, 304, 305.1–9 | F11–19 |
| Suicides and deaths from undetermined intent | E95, E98 | X60-X84, Y10-Y34 |
| Obesity^d^ | 278A, 278X | E65, E66 |
| Cardiovascular disease (CVD) |  |  |
| Coronary heart disease | 410–414 | I20–I25 |
| Ischemic stroke | 434, 436 | I63–I64 |
| Hypertension^e^ | 401–405 | I10–I15 |
| Diabetes mellitus^e^ | 250 | E10–E14 |
| Valvular disease | 394–397, 424 | I05–I09, I33–I39 |
| Atrial fibrillation | 427.3 | I48 |
| Cardiomyopathy | 425 | I42, I43 |
| Malignancy | 140–208 | C00–C97 |

Abbreviations: ICD, International Classification of Diseases

^a^ ICD-9 was in use from 1987 to 1996, and ICD-10 since 1997 and onwards.

^b^ Comorbidity was defined as having one of the above stated ICD codes registered in the Patient Register prior to or at study baseline.

^c^ Depressive disorders was defined either through diagnosis in the Patient Register or trough dispensed antidepressant medication (ATC-code N06A)

^d^ All individuals with one of these ICD codes for obesity registered in the Patient Register between 1987 and 2001 were excluded from the study sample.

^e^ Hypertension and diabetes was defined either through a diagnosis in the Patient Register or trough dispensed antihypertensive or antidiabetic medication. Full definition below.

**Table S3**. Hazard ratios for all outcomes, divided into three time-periods^a^, comparing gastric bypass patients and non-operated patients with obesity with non-obese population controls.

|  | **First period 0–2 years** | | **Second period 2–6 years** | | **Third period 6–10 years** | |
| --- | --- | --- | --- | --- | --- | --- |
| **Outcomes** | **N (event)** | **Hazard ratios (95% CI)** | **N (event)** | **Hazard ratios (95% CI)** | **N (event)** | **Hazard ratios (95% CI)** |
| **Depressive disorders (diagnosis) and Neurotic, stress-related and somatoform disorders** | | | | | | |
| Gastric bypass patients | 16,932 (410) | 1.62 (1.38-1.91) | 13,537 (589) | 2.38 (2.07-2.73) | 3839 (150) | 2.38 (2.07-2.73) |
| Non-operated patients w obesity | 25,698 (690) | 2.02 (1.75-2.33) | 20,666 (924) | 2.29 (2.02-2.60) | 11,082 (394) | 2.29 (2.02-2.60) |
| Non-obese population controls | 66,007 (840) | 1 (Reference) | 53,060 (1018) | 1 (Reference) | 27,850 (562) | 1 (Reference) |
| **Depressive disorders (diagnosis)** | | | | | | |
| Gastric bypass patients | 16,932 (221) | 1.88 (1.50-2.36) | 14,210 (335) | 2.19 (1.83–2.63) | 4167 (100) | 2.19 (1.83–2.63) |
| Non-operated patients w obesity | 26,966 (333) | 1.83 (1.48-2.27) | 22,054 (633) | 2.44 (2.09-2.86) | 12,159(364) | 2.44 (2.09-2.86) |
| Non-obese population controls | 67,640 (394) | 1 (Reference) | 54,752 (552) | 1 (Reference) | 29,160(387) | 1 (Reference) |
| **Neurotic, stress-related and somatoform disorders** | | | | | | |
| Gastric bypass patients | 24,054 (872) | 1.58 (1.38-1.80) | 19,565 (963) | 2.07 (1.83-2.33) | 5224 (192) | 2.07 (1.83-2.33) |
| Non-operated patients w obesity | 34,551 (969) | 1.59 (1.40-1.81) | 27,066 (1077) | 1.79 (1.58-2.01) | 13,483(408) | 1.79 (1.58-2.01) |
| Non-obese population controls | 75,648 (935) | 1 (Reference) | 60,174 (1118) | 1 (Reference) | 30,562 (538) | 1 (Reference) |
| **Anti-depressant medication** |  |  |  |  |  |  |
| Gastric bypass patients | 16,156 (1500) | 2.01 (1.84-2.20) | 12,301 (260) | 2.19 (2.0-2.39) | 2674 (244) | 2.19 (2.0-2.39) |
| Non-operated patients w obesity | 19,853 (1532) | 1.77 (1.61–1.94) | 14,617 (231) | 2.07 (1.9-2.26) | 5432 (543) | 2.07 (1.9-2.26) |
| Non-obese population controls | 52,813 (2075) | 1 (Reference) | 39,884 (382) | 1 (Reference) | 15,437 (806) | 1 (Reference) |
| **Alcohol-related disorders** |  |  |  |  |  |  |
| Gastric bypass patients | 27,264 (324) | 3.87 (3.14-4.76) | 22,406 (587) | 4.94 (4.07-5.98) | 6138 (134) | 4.94 (4.07-5.98) |
| Non-operated patients w obesity | 39,125 (274) | 1.15 (0.90-1.48) | 31,157 (283) | 1.38 (1.10-1.74) | 15,739 (129) | 1.38 (1.10-1.74) |
| Non-obese population controls | 79,646 (196) | 1 (Reference) | 63,146 (285) | 1 (Reference) | 32,380 (156) | 1 (Reference) |
| **Other substance use disorders** |  |  |  |  |  |  |
| Gastric bypass patients | 27,264 (324) | 2.37 (1.86-3.03) | 22,654 (376) | 2.67 (2.15-3.30) | 6314 (93) | 2.67 (2.15-3.30) |
| Non-operated patients w obesity | 39,125 (277) | 1.62 (1.26-2.10) | 31,069 (356) | 1.66 (1.33-2.07) | 15,719 (198) | 1.66 (1.33-2.07) |
| Non-obese population controls | 79,646 (196) | 1 (Reference) | 63,843 (270) | 1 (Reference) | 32,733 (180) | 1 (Reference) |
| **Suicide** |  |  |  |  |  |  |
| Gastric bypass patients | 28,204 (37) | 3.42 (1.57-7.46) | 23,684 (42) | 1.92 (1.01-3.65) | 6751 (19) | 1.92 (1.01-3.65) |
| Non-operated patients w obesity | 40,827 (34) | 1.64 (0.71–3.77) | 32,637 (38) | 1.29 (0.68-2.46) | 16,850 (22) | 1.29 (0.68-2.46) |
| Non-obese population controls | 80,800 (27) | 1 (Reference) | 64.918 (28) | 1 (Reference) | 33,601 (9) | 1 (Reference) |

CI: confidence intervals

Hazard ratios and 95% CI for <2 years, 2–6 years and >6–10 years of follow-up.

Model adjusted for age, sex, education level, marital status, and baseline depressive disorders (diagnosis), neurotic, stress-related and somatoform disorders, anti-depressant medication, alcohol-related disorders, and other substance abuse.

**Table S4**. Hazard ratios for all outcomes, divided into three time-periods^a^, comparing gastric bypass patients with non-operated patients with obesity.

|  | **First period 0–2 years** | | **Second period 2–6 years** | | **Third period 6–10 years** | |
| --- | --- | --- | --- | --- | --- | --- |
| **Outcomes** | **N (event)** | **Hazard ratios (95% CI)** | **N (event)** | **Hazard ratios (95% CI)** | **N (event)** | **Hazard ratios (95% CI)** |
| **Depressive disorders (diagnosis) and Neurotic, stress-related and somatoform disorders** | | | | | | |
| Gastric bypass patients | 16,932 (410) | 1.62 (1.38-1.91) | 13,537 (589) | 2.38 (2.07-2.73) | 3839 (150) | 2.38 (2.07-2.73) |
| Non-operated patients w obesity | 25,698 (690) | 2.02 (1.75-2.33) | 20,666 (924) | 2.29 (2.02-2.60) | 11,082 (394) | 2.29 (2.02-2.60) |
| **Depressive disorders (diagnosis)** | | | | | | |
| Gastric bypass patients | 16,932 (221) | 1.88 (1.50-2.36) | 14,210 (335) | 2.19 (1.83–2.63) | 4167 (100) | 2.19 (1.83–2.63) |
| Non-operated patients w obesity | 26,966 (333) | 1.83 (1.48-2.27) | 22,054 (633) | 2.44 (2.09-2.86) | 12,159(364) | 2.44 (2.09-2.86) |
| **Neurotic, stress-related and somatoform disorders** | | | | | | |
| Gastric bypass patients | 24,054 (872) | 1.58 (1.38-1.80) | 19,565 (963) | 2.07 (1.83-2.33) | 5224 (192) | 2.07 (1.83-2.33) |
| Non-operated patients w obesity | 34,551 (969) | 1.59 (1.40-1.81) | 27,066 (1077) | 1.79 (1.58-2.01) | 13,483(408) | 1.79 (1.58-2.01) |
| **Anti-depressant medication** |  |  |  |  |  |  |
| Gastric bypass patients | 16,156 (1500) | 2.01 (1.84-2.20) | 12,301 (260) | 2.19 (2.0-2.39) | 2674 (244) | 2.19 (2.0-2.39) |
| Non-operated patients w obesity | 19,853 (1532) | 1.77 (1.61–1.94) | 14,617 (231) | 2.07 (1.9-2.26) | 5432 (543) | 2.07 (1.9-2.26) |
| **Alcohol-related disorders** |  |  |  |  |  |  |
| Gastric bypass patients | 27,264 (324) | 3.87 (3.14-4.76) | 22,406 (587) | 4.94 (4.07-5.98) | 6138 (134) | 4.94 (4.07-5.98) |
| Non-operated patients w obesity | 39,125 (274) | 1.15 (0.90-1.48) | 31,157 (283) | 1.38 (1.10-1.74) | 15,739 (129) | 1.38 (1.10-1.74) |
| **Other substance use disorders** |  |  |  |  |  |  |
| Gastric bypass patients | 27,264 (324) | 2.37 (1.86-3.03) | 22,654 (376) | 2.67 (2.15-3.30) | 6314 (93) | 2.67 (2.15-3.30) |
| Non-operated patients w obesity | 39,125 (277) | 1.62 (1.26-2.10) | 31,069 (356) | 1.66 (1.33-2.07) | 15,719 (198) | 1.66 (1.33-2.07) |
| **Suicide** |  |  |  |  |  |  |
| Gastric bypass patients | 28,204 (37) | 3.42 (1.57-7.46) | 23,684 (42) | 1.92 (1.01-3.65) | 6751 (19) | 1.92 (1.01-3.65) |
| Non-operated patients w obesity | 40,827 (34) | 1.64 (0.71–3.77) | 32,637 (38) | 1.29 (0.68-2.46) | 16,850 (22) | 1.29 (0.68-2.46) |

CI: confidence intervals

Hazard ratios and 95% CI for <2 years, 2–6 years and >6–10 years of follow-up.

Model adjusted for age, sex, education level, marital status, and baseline depressive disorders (diagnosis), neurotic, stress-related and somatoform disorders, anti-depressant medication, alcohol-related disorders, and other substance abuse.
